# Artificial intelligence (AI) in action: a cross-continental survey of AI adoption among field epidemiology fellows in Canada, the United States, and Europe, 2024

**DOI:** 10.2807/1560-7917.ES.2026.31.26.2500677

**Published:** 2026-07-02

**Authors:** Courtney R Smith, Natalie Girin, Kathleen Laberge, Seth Manthey, Pasha Marcynuk, Eric Pevzner, Adam Roth, Louis Wong

**Affiliations:** 1 Canadian Field Epidemiology Program, Public Health Agency of Canada; 100 Colonnade Road, Ottawa, Ontario, Canada; 2 Fellowship Programme Section, Surveillance Preparedness and Response, European Centre for Disease Prevention and Control, Stockholm, Sweden; 3 Epidemic Intelligence Service, Centers for Disease Control and Prevention; 1600 Clifton Road, Atlanta, Georgia, United States

**Keywords:** Artificial intelligence, AI, field epidemiology, field epidemiology training programs

## Abstract

**BACKGROUND:**

Technologies available for applied public health practice are rapidly evolving. Artificial intelligence (AI) tools have become more accessible, however, their utilisation by field epidemiologists has not been described.

**AIM:**

We explored AI use among Canadian, European, and United States (US) Field Epidemiology Training Program (FETP) fellows.

**METHODS:**

Fellows in the three programmes (n = 220) were invited to an online survey available from 25 November 2024 to 5 December 2024. Quantitative and qualitative data were collected concerning the AI platforms fellows used, AI applications, comfort level when applying AI, use frequency, impact on work, barriers, ethical concerns, AI policy/guideline availability, and AI training.

**RESULTS:**

There were 105 (48%) survey respondents: 13, 56 and 36 in the Canadian, US and European FETPs respectively. The proportion of respondents who reported applying AI for work was 66% (69/105) overall, and highest among respondents of the European programme (32/36). The most adopted platform was ChatGPT (60/69; 87%), and the most common application was analytical coding assistance (63/69; 91%). Among AI users, most felt somewhat comfortable (34/69; 49%) or comfortable (24/69; 35%) with AI and reported weekly (29/69; 42%) or daily (21/69; 30%) use. Generally, AI’s reported impact was positive. Challenges included technical difficulties, accuracy concerns, bias introduction, uncertainty regarding permissible uses, and data privacy issues. Among respondents, 47% (49/105) affirmed having AI policies/guidelines in their institutions and 20% (21/105) had received AI training.

**CONCLUSION:**

To further incorporate AI use among its fellows, FETPs should monitor AI’s evolving role, and provide guidance and adapt curricula accordingly.

**Key public health message**

**What did you want to address in this study and why?**

While artificial intelligence (AI) tools have become more readily available, their use by practicing field epidemiologists and integration into Field Epidemiology Training Programs (FETPs) has not been studied. The current survey aimed to fill that gap by questioning field epidemiology fellows in Canada, Europe and the United States on how they engaged with AI tools in their routine work, on the concerns they experienced, and if they received training.

**What have we learnt from this study?**

Two-thirds of the 105 survey participants reported AI use, with the highest rate (32/36) among European FETP fellow respondents. ChatGPT was the most used platform, and AI was mainly employed to support analytical coding and data analysis. Despite growing comfort with AI, accuracy, bias, and ethical concerns persisted, calling for structured guidance. Only one in five fellows had received training, highlighting AI education needs.

**What are the implications of your findings for public health?**

As AI becomes more embedded in public health practice, FETPs must include education allowing fellows to develop and maintain adequate AI competencies for current practice and anticipated needs or requirements. Addressing technical, interpretation, ethical, and privacy concerns will ensure effective and responsible use. This study supports implementing and continuously adapting targeted training and standardised guidelines for AI integration in epidemiology.

## Introduction

The public health landscape is evolving, as are the technologies available for use by public health practitioners. Recently, artificial intelligence (AI) tools have become easily accessible and their use in public health is increasing. In the current report, AI refers to a broad range of technologies that can perform tasks typically associated with the cognitive functions of humans, including recognition, learning, and logical reasoning [1]. Although the full potential of AI in applied public health remains untapped, its applications are promising, and public health practitioners are already using AI for disease surveillance, risk assessment, pathogen classification, outbreak modelling, outbreak detection, source identification, scientific communication, and even epidemiological study design [2-7]. However, concerns, challenges, and shortcomings associated with the application of AI in public health have arisen. These include, but are not limited to the introduction of bias, accuracy of responses, ethics, data privacy, and gaps in the expertise needed to use AI and interpret its results [2,8-11].

Field Epidemiology Training Programs (FETPs) are specialised programmes dedicated to epidemiology education and application. Around the world, these training programmes serve to build capacity in public health agencies that operate locally, regionally, nationally and at international level [12]. Across programmes, trainees go by different titles; for the purposes of this paper, they will be referred to as field epidemiology fellows. They work within their assigned jurisdictions for limited periods on a range of topics, across both communicable and non-communicable diseases, and gain experience in outbreak investigation, disease surveillance, public health programme development, epidemiological analysis, and in responding to urgent public health needs. At time of writing in 2026, approximatively 90 FETPs were operating within 200 territories and countries around the world [12]. These programmes are categorised based on the qualifications required of applicants and the duration of training. The three categories are basic or ‘frontline’ (3 months, part-time, and intended for surveillance officers), ‘intermediate’ (9–12 months, part-time, and intended for junior epidemiologists), and ‘advanced’ (2 years, full time, and intended for graduate-level epidemiologists). Advanced programmes have fellows spend about 75% of their time learning through service at a placement site, and around 25% on formal training curriculum in a physical or virtual classroom setting. Fellows are assigned to local, regional, or national public health agencies where they work under the supervision of experienced epidemiologists.

Field epidemiology differs from more academic or analytic forms of epidemiology in ways that have direct implications for the adoption of AI. Field epidemiologists are frequently engaged in real-time decision-making during public health emergencies, often working with incomplete and rapidly evolving data. As such, their work places a premium on judgment and interpretation, and the ability to communicate uncertainty. The objective of FETPs is for field epidemiology fellows to develop technical capabilities in applied epidemiology, while also improving their aptitude to communicate information and building their professional judgment. While AI tools may enhance efficiency and support certain technical tasks and scientific communication products, their use within training settings raises an important consideration, namely, to avoid overreliance on AI at the expense of human learning and critical thinking. As leaders of FETPs, we believe that AI could be adopted within the context of training programmes, prioritising the development of skills and competency to interpret and use data for decision-making, and ultimately, public health action.

While field epidemiologists and public health organisations stand to benefit from the integration of AI into public health practice [8], limited information is available regarding the use of AI among field epidemiologists in their day-to-day work. Three advanced FETPs – the Canadian Field Epidemiology Program (CFEP) [13]; the United States (US) Centers for Disease Control and Prevention’s (CDC) Epidemic Intelligence Service (EIS) [14]; and the European Centre for Disease Prevention and Control (ECDC) Fellowship Programme [15] – collaborated to learn about the use of AI among their fellows. The study objectives were to understand the use of AI among advanced-level field epidemiology fellows in Canada, the US, and Europe and summarise findings. In particular, this project aimed to investigate how fellows engaged with AI, how AI impacted their work, the barriers and ethical concerns they faced, and if they were aware of AI policies or guidelines at their institutions. Moreover, whether fellows received or needed AI training was also explored. The overall goal was to leverage findings for guiding integration of AI into use by FETPs worldwide.

## Methods

### Survey development and delivery

The cross-sectional survey was developed in collaboration across the three advanced FETPs. An iterative process was used in which potential questions were proposed and refined to ensure they reflected programme priorities and information needs at the time of survey development. Questions were designed to address identified gaps in knowledge regarding the extent to which field epidemiology fellows were using AI tools, and the purposes and impacts of these tools. The questions further explored how fellows perceived barriers and concerns regarding the use of AI, as well as their awareness of AI institutional guidelines or policies and reception or need of training. The primary intent of the survey was to assess current practices and inform future programme development, training, and support.

The survey, which is described in the Supplement S1, consisted of a combination of 20 open- and closed-ended questions, capturing both quantitative and qualitative data. Responses were kept anonymous, and survey questions were designed in a neutral tone to reduce response bias, as well as social desirability bias. The survey included six sections. While the first section was about the (i) training programme details (i.e. specific FETP programme and year of programme), the subsequent sections aimed to understand (ii) how fellows engage with AI and (iii) its effects on their work, as well as (iv) the barriers and ethical concerns fellows face with regards to AI use and (v) their knowledge of institutional policies and guidelines concerning AI. The last section was about (vi) AI-associated training. The survey was pilot tested internally by the management teams and staff of the FETPs. The survey was hosted on Microsoft Forms (Microsoft Corporation, Redmond, Washington, US) and was available from 25 November 2024 to 5 December 2024, in English. Within the survey, AI was defined as a broad range of technologies that can perform tasks typically associated with the cognitive functions of humans (e.g. recognition, learning, logical reasoning).

All fellows who were currently enrolled in the three advanced FETPs at the time of the survey were eligible to complete the survey. This comprised a total of 220 field epidemiology fellows from the Canadian (n = 13), US (n = 135) and the European (n = 72) FETPs. The European FETP included both paths of intervention epidemiology (EPIET) and public health microbiology (EUPHEM) [15], as well as fellows in the German EPIET-associated programme, Postgraduate Training for Applied Epidemiology (PAE). Invitations and email reminders were sent by each respective FETP. For European fellows, communication about the survey and reminders were sent by announcements on their dedicated cohort pages that are located on the ECDC learning portal. Survey completion was encouraged, but not mandatory. All questions were optional and all responses were anonymous.

### Analysis

For closed-ended survey questions, data were exported from Microsoft Forms and descriptive analyses were performed using Microsoft Office 365 Excel. Overall responses, responses by programme (Canadian, US, and European FETP), and by programme year (first year or second year) were considered. To test for differences in reported AI use between programmes and by programme year, chi-square tests were performed with an alpha level of 0.05. Open-ended survey responses on AI impact, barriers, ethical concerns, and training needs were analysed thematically to contextualise the quantitative findings. A single coder reviewed all responses and grouped them into overarching themes, which were then shared with coauthors along with the raw data for further checking. This approach ensured that the reported themes accurately reflected participants’ feedback while aligning with the programmatic aims of the survey.

## Results

Of the 220 field epidemiology fellows, 105 (48%) completed the survey. Of these, 13 were from the Canadian programme (response rate: 13/13, 100%), 56 were from the US programme (response rate: 56/135; 41%), and 36 were from the European programme (response rate: 36/72; 50%). Overall, among the 105 respondents 53 (50%) were enrolled in the first year of their respective FETP and 52 (50%) were in the second year (Table).

**Table:** Artificial intelligence survey results across the Canadian, United States and European Field Epidemiology Training Programs, 2024 (n = 105 participants)

Aspects covered by the survey	Programme			Total n = 105				
	Canadian n = 13	US n = 56	European n = 36					
	Proportion	Proportion	Proportion	Number		Denominator		%
**Year of FETP programme**								
First year	8/13	23/56	22/36	53		105		50
Second year	5/13	33/56	14/36	52		105		50
**How FETP fellows engage with AI**								
**Use of AI in work as FETP fellow**								
Yes	7/13	30/56	32/36	69	105		66	
No	6/13	26/56	4/36	36	105		34	
**Frequency of use of AI**								
Daily	1/7	11/30	9/32	21	69		30	
Weekly	2/7	10/30	17/32	29	69		42	
Monthly	0/7	0/30	1/32	1	69		1	
On occasion	4/7	9/30	5/32	18	69		26	
**Reported comfort with AI use**								
Uncomfortable	1/7	1/30	1/32	3	69		4	
Somewhat comfortable	3/7	15/30	16/32	34	69		49	
Comfortable	2/7	11/30	11/32	24	69		35	
Very comfortable	1/7	3/30	4/32	8	69		12	
**AI platform use^a^**								
ChatGPT	6/7	24/30	30/32	60	69		87	
Institution-specific platform	0/7	14/30	3/32	17	69		25	
Microsoft Bing Co-Pilot	1/7	1/30	7/32	9	69		13	
Gemini or Perplexity	3/7	3/30	2/32	8	69		12	
Claude	1/7	2/30	1/32	4	69		6	
Other	0/7	1/30	4/32	5	69		7	
**Applications of AI^a^**								
Troubleshoot coding errors	7/7	27/30	29/32	63	69		91	
Write code	7/7	23/30	22/32	52	69		75	
Work efficiencies e.g. drafting emails, note-taking	3/7	10/30	16/32	29	69		42	
Gather background information on a public health or other topic	4/7	8/ 30	9/32	21	69		30	
Draft text for abstracts and other written deliverables	2/7	10/30	8/32	20	69		29	
Generate suggestions for analytic methods	1/7	11/30	7/32	19	69		28	
Generate epidemiological study questions	0/7	3/30	1/32	4	69		6	
Generate images for presentations	0/7	2/30	2/32	4	69		6	
Other	1/7	2/30	9/32	12	69		17	
**Reported barriers to AI use**								
Yes	1/7	11/30	16/32	28	69		41	
No	6/7	19/30	16/32	41	69		59	
**Encountered ethical or privacy issues with AI use**								
Yes	2/7	3/30	12/32	17	69		25	
No	5/7	27/30	20/32	52	69		75	
**Availability of AI policy or guideline at institution**								
Yes	6/13	35/56	8/36	49	105		47	
No	0/13	1/56	11/36	12	105		11	
Unsure	7/13	20/56	17/36	44	105		42	
**Received AI training**								
Yes	3/13	13/56	5/36	21	105		20	
No	10/13	43/56	31/36	84	105		80	

AI: artificial intelligence; ChatGPT: Chat-Generative Pre-training Transformer; FETP: Field Epidemiology Training Programs; US: United States.

**^a^** Fellows were permitted to select multiple responses; the results are not mutually exclusive.

### Engagement with artificial intelligence

#### Overall use of artificial intelligence

Across all 105 respondents, 66% reported using AI in their work as a field epidemiology fellow (Table). Reported AI use was greater among respondents in the European programme (32/36) than the US (30/56) or Canadian (7/13) programmes (p = 0.0015). Slightly more first-year fellows reported using AI (37/53) than second-year fellows (32/52)*;* however, this difference was not statistically significant (p = 0.80).

Across all respondents, 36 (34%) indicated that they do not use AI in their work. Of these fellows, 34 provided additional qualitative responses explaining their reasons. Reported reasons for not using AI included a perception that AI is unnecessary or not useful for their current projects, a lack of trust in AI-generated results, and limited experience with AI. Respondents indicated that they felt reasons for not using AI might be addressed with further training regarding its potential applications in public health. Some respondents expressed moral or ethical concerns, including problems associated with data scraping or privacy. A few respondents reported concerns about environmental effects of AI technologies. Other respondents were uncertain about whether the use within their organisation (i.e. placement site) was permissible. 

#### Frequency of use

Among the 69 fellows reporting using AI in their work, 30% reported daily use (Table), 42% reported weekly use, and 26% occasional use. Frequency of use seemed to vary by programme, with daily use reported most frequently among respondents of the US programme (11/30), weekly use reported most frequently among European respondents (17/32), and occasional use reported most frequently among Canadian programme respondents (4/7).

#### Comfort with artificial intelligence use

Among the 69 fellows who used AI, the highest proportion of fellows reported feeling somewhat comfortable using AI in their work (49%; Table), followed by those who were comfortable (35%), very comfortable (12%), and uncomfortable (4%). Trends in comfort were similar across all three FETPs.

#### Platforms used

ChatGPT (OpenAI, San Francisco, California, US) was the most frequently reported AI platform used by the field epidemiology fellows (87%; Table), followed by institution-specific AI platforms (25%), Microsoft Bing Co-Pilot (13%), Gemini (Google DeepMind, Mountain View, California, US) or Perplexity (Perplexity AI, San Francisco, California, US) (12%), and Claude (Anthropic, San Francisco, California, US) (6%). Overall, 7% of fellows reported AI platforms not included in the survey; these platforms included Phind (Hello Cognition, San Francisco, California, US), Grammarly (Grammarly, San Francisco, California, US), Rayyan (Rayyan Systems, Boston, Massachusetts, US), Covidence (Veritas Health Innovation, Melbourne, Australia), and DeepL (DeepL SE, Cologne, North Rhine-Westphalia, Germany). Because respondents were permitted to select multiple AI platforms, the results are not mutually exclusive. Trends in platforms used were broadly similar across FETPs, except for the US programme where about half (14/30) of respondents reported the highest use of institution-specific AI platforms.

#### Applications of artificial intelligence

The most frequently reported applications of AI were troubleshooting coding errors (91%; Table), writing code (75%), and improving work efficiencies (e.g. drafting emails or note taking) (42%). Other reported uses included gathering background information regarding public health topics (30%), drafting text as a starting point for abstracts or other written work (29%), generating suggestions for analytic methods (28%), generating images for presentations (6%), and generating epidemiological study questions (6%). Overall, 17% of respondents reported using applications for AI not included in the survey. These uses included grammar and spelling checks, summarising large texts, enhancing writing quality, and translating text. The results were not mutually exclusive as respondents were permitted to select multiple AI applications. Troubleshooting coding errors and writing code were the top two applications across all programmes. The third most common AI application varied by programme, with respondents from the Canadian programme using AI for gathering background information (4/7), respondents from the European programme using AI for work efficiencies (16/32), and respondents from the US programme using AI for generating suggestions for analytic methods (11/30).

### Effects of artificial intelligence on daily work

Of the 69 fellows who reported using AI, 56 (81%) provided additional qualitative responses on how AI has affected their work. Responses fell into three key themes. First, the majority of respondents highlighted AI’s positive effect on coding efficiency. For example, AI reduced the time spent troubleshooting coding errors, aided in learning new coding techniques, simplified existing code, facilitated code transfer across different coding software programmes, and was helpful for generating ideas for data analysis.

Second, some fellows noted AI's positive effects on both the efficiency and quality of their writing. Respondents reported that AI reduced writing time, improved the quality of drafts, assisted in situations where word count needed to be reduced, and was helpful with grammar and phrasing, particularly for those with English as a second language.

Third, some respondents cited AI's positive contribution to efficiencies in their daily work. Examples included using AI for drafting emails, writing meeting minutes, organising project timelines, and aiding in the understanding of new topics or conducting background research and summarising information.

### Barriers and ethical concerns

Overall, 28 of the 69 fellows (41%; Table) reported barriers to using AI in their work, of whom 27 provided additional qualitative responses to describe the types of barriers they faced. Reported barriers included technical challenges, access and permission limitations, and paywall restrictions. Certain free AI platforms were criticised for imposing query limits or not saving previous answers. Additional barriers reported by field epidemiology fellows included concerns with the accuracy and reproducibility of AI outputs, and uncertainty with what was considered acceptable use at their institution or within their FETP. Certain reported barriers also intersected with ethical concerns, which are summarised as follows.

Overall, 17 field epidemiology fellows (25%; Table) expressed ethical concerns regarding the use of AI. Of these 17 fellows, 13 provided additional qualitative responses on the types of ethical concerns they faced, which included data privacy, storage of data, and how data inputted into AI systems might be used. Further ethical concerns were the environmental effects of AI, and some respondents raised concerns about the introduction of bias through AI. Some respondents were concerned about becoming over reliant on using AI in their work, particularly within the context of a training programme.

### Availability of institutional policies or guidelines

In total, 47% (Table) of respondents were aware of their institutions’ AI policy or guidelines; however, 42% were unsure whether such a policy or guidelines existed. Approximately 11% of respondents reported that their institution did not have an AI policy or guideline. Among 49 respondents who knew their institution had a guideline, 33 were aware of where to find it.

### Training regarding the use of artificial intelligence

Only 20% of fellows (Table) reported receiving formal or informal training regarding the use of AI. In their feedback, 76 respondents outlined multiple training needs. Many expressed a desire for hands-on instruction regarding how to integrate AI into public health practices, including selecting the most appropriate AI tools for specific tasks. They also requested training in practical applications, such as using AI for coding, data analysis, scientific writing, data visualisation, and table creation. An interest in finding out how to leverage AI for surveillance and outbreak detection was reported, along with understanding relevant use cases (e.g. how public health professionals and institutions have used AI for surveillance or outbreak detection in real life settings) and learning effective prompt strategies when using AI. Some respondents highlighted the importance of foundational knowledge about AI. Respondents wanted to know how AI works so they could more fully understand its biases and limitations. Furthermore, a request for instruction on how to avoid common pitfalls and recognise errors was reported. Ethical considerations involving the use of AI in public health were also identified as a crucial area for training.

## Discussion

In this report regarding use of AI among field epidemiology fellows across three large FETPs from Canada, the US, and Europe, approximately two-thirds of fellows were using AI in their work. The most used platform was ChatGPT, and the primary application was for coding assistance. Most fellows using AI felt comfortable in its use and reported using it daily or weekly. However, barriers, ethical concerns, and a need for formal training was expressed. Although other work has summarised the potential applications of AI among field epidemiologists [8], this is the first study, to the best of our knowledge, that has surveyed current fellows in field epidemiology regarding AI practices.

Several of the main findings warrant deeper exploration and discussion, regarding the implications for the field of public health and epidemiology. First, differences in AI usage among the multiple FETPs suggest that organisational context and programme-specific factors might influence the adoption and integration of AI tools. Fellows from the European FETP reported a higher level of AI use (32/36), compared with those from the US (30/56) and Canadian programmes (7/13). These differences might be a result of barriers in the US and Canadian programmes, such as a lack of familiarity with AI tools or institutional restrictions. Cultural differences in AI acceptance or promotion may also play role. Second, among users of AI, the most common use by far was for troubleshooting coding errors and writing code. Capitalising on AI for coding assistance has been well documented in the fields of computer programming [16,17] and software engineering [18], and perhaps presents itself as the most immediately accessible opportunity for the integration of AI into the domain of field epidemiology. Third, concerns about barriers and ethical considerations with AI were present among 41% and 25% of field epidemiology fellows who reported using AI, respectively. Similar concerns were expressed by certain fellows not using AI, as some of the reasons for avoiding using it entirely. The barriers and concerns raised, such as data privacy, AI's accuracy and reproducibility, environmental effects, and introduction of bias, underscore the complexity of AI adoption in applied public health. These concerns highlight the need for FETPs to address the technical and epistemic dimension of AI use through training and education, and ethical dimensions of AI use through renewed guidelines, which are both discussed as follows.

Based on the findings of this study, the authors recommend that field epidemiology and other public health training programmes consider adapting their training curricula to incorporate AI, to benefit from increased efficiencies that can be derived from its use. Leaders of training programmes can equip the fellows with the training required to build the necessary skills and knowledge to apply AI in their work. Given the rapid pace of AI development, curricula should integrate both foundational AI literacy, explaining how AI works, limitations, potential biases and ethical implications, and practical applications tailored to the specific needs of field epidemiologists. Possible recommendations for training might include hands-on workshops, real-world case studies, and access to AI tools and platforms. The FETPs need to ensure learners gain experience using AI in foundational epidemiological tasks like coding, study design, data analysis, scientific writing, and topics such as surveillance and outbreak detection. Existing training courses can be modified to incorporate AI into their learning objectives and practice using AI as a support tool while learning foundational skills and applying critical thinking. Alternatively, education and capacity building in AI can extend beyond formal FETP curricula and may be supported through a broader ecosystem that includes universities, professional societies, and alumni and professional networks. The focus of training, as highlighted by others, should be the use of AI to augment one’s skills, not replace them [8]. Additionally, FETPs should share continuing education opportunities to fellows both during and after completion of their fellowship, such as webinars and online courses [19,20], to allow current trainees and graduates to stay up to date on new AI developments. A key challenge in this process will be the potential lag between the adoption of AI technologies by fellows and their supervisors or trainers. To address this, FETPs can adopt a train-the-trainer model, ensuring that those in supervisory roles not only understand AI but are also capable of mentoring and guiding trainees in responsible use of what should be a complementary technology. This approach would create a feedback loop, where both trainers and trainees can learn from each other’s experiences, ensuring that the programme stays on the cutting edge of AI integration. Lastly, FETPs should collaborate with AI experts, public health organisations, academic institutions, professional societies, and alumni networks to continuously refine curricula and ensure that their training programmes remain relevant in an ever-evolving technological landscape.

As AI continues to evolve, establishing clear and comprehensive guidance for use in FETPs presents a considerable challenge. The fast pace of technological advancement means that what is considered appropriate or effective today can be outdated tomorrow. However, the development of this guidance is necessary to balance between harnessing the potential and the responsible use of AI. Current guidelines exist within the federal government in Canada [21-24], at the European Union level [25], and in the US [26]. The guidance is broadly applicable but lacks specific use-cases for applications in public health and applied epidemiology, which have unique considerations, as summarised previously. To address both applicability and the rapid evolution of AI, the ECDC Fellowship Programme maintains a living working document that guides fellows on the ‘do’s and don’ts’ of AI use [27], grounded in the guiding principles of the EU AI act [25]. The authors propose three primary considerations in the development of similar AI guidance within FETPs and other public health programmes. 

First, a clear tension exists between the FETP mandate to teach the principles of applied epidemiology, and an overreliance on AI, which might hinder learning. The authors believe training programmes should prioritise skill development and critical thinking, while ensuring that AI does not overshadow foundational learning or replace human judgment. Second, in the context of public health, risks associated with data privacy, accuracy, and bias should be at the forefront of any guidance. Field epidemiologists should be educated on the potential pitfalls of AI, including how AI can inadvertently introduce bias into research findings or compromise data privacy and confidentiality. Third, a need exists to assess how AI tools align with broader public health goals. For instance, considerations can be incorporated regarding AI's environmental effects, for which evidence exists for both positive [28,29] and negative [30] effects. Overall, clear guidelines are necessary to help fellows navigate these complexities. We need guidelines that ensure AI is used effectively, ethically, and in a manner that supports and does not undermine the goal of FETPs.

Multiple limitations should be considered when interpreting the findings of this survey. First, although the survey included fellows from three FETPs across two continents, it did not include all FETPs globally; this affects the generalisability of the results. Findings should be interpreted as reflective of survey participants rather than all fellows across FETPs. Second, the response rate for the survey was 48%, which may introduce nonresponse bias, as those who completed the survey might differ in their experiences with AI from those who did not. If those more interested in or familiar with AI were more likely to respond, this could have potentially biased the results. Responses were kept anonymous, and survey questions were designed in a neutral tone to reduce response bias, as well as social desirability bias; however, nonresponse bias might still be present. The relatively small number of respondents also prevented more granular analyses, which may have been informative. In addition, while all field epidemiology fellows included in the survey are proficient in English, administering the survey in English may have excluded nuanced perspectives from individuals for whom English is not a first language. Third, the rapidly evolving nature of AI technologies raises questions about the long-term relevance of these findings. The authors acknowledge that this survey provides a snapshot of AI use at a specific point in time. Additionally, the survey only gathered perspectives from the fellows themselves, not from their placement sites, where views and experiences with AI might differ. This might be a valuable area for future exploration. Fourth, the reliance on self-reported data means that responses might be subject to social desirability bias or inaccuracies in recalling AI use. Approximately one-third of fellows were not using AI, suggesting there might be underlying factors that were not explored in this survey. Investigating this gap might offer valuable insights and represent a potential next step for this work. Lastly, the survey did not collect information on sex, racial identity, ethnicity, or age, and thus differences in AI use across these groups could not be explored.

## Conclusion

The results of this study provide insight into the adoption of AI tools among field epidemiology fellows in Canada, the US, and Europe. The findings underscore the need for FETPs to more deeply understand the evolving role of AI in public health. To remain relevant and effective, these programmes should adapt their curricula and guidance to better incorporate AI technologies. This will not only equip future epidemiologists with the skills needed to leverage AI in their work but also ensure that the next generation of public health professionals is prepared for the challenges and opportunities presented by these rapidly advancing tools.

## Data Availability

Data from this study are not publicly available. Please contact the corresponding author (CRS) for additional information on the data.

## Supplementary Data

168000 Supplement S1
