# Supplementary material for: Artificial intelligence (AI) in action: a cross-continental survey of AI adoption among field epidemiology fellows in Canada, the United States, and Europe, 2024
Source: Euro Surveill. 2026 Jul 2;31(26):2500677. doi: 10.2807/1560-7917.ES.2026.31.26.2500677 (PMC13332366; doi:10.2807/1560-7917.ES.2026.31.26.2500677)
Supplement: Supplement S1 [file 25-00677_Supplementary_Material.pdf]

S1. Artificial Intelligence Survey for Field Epidemiology Fellows of the Europe, United States, and Canada Field Epidemiology Training Programs

Disclaimer: This supplementary material is hosted by Eurosurveillance as supporting information alongside the article "Artificial Intelligence in Action: A Cross-Continental Survey of AI Adoption Among Field Epidemiology Fellows in Canada, the United States, and Europe, 2024" on behalf of the authors, who remain responsible for the accuracy and appropriateness of the content. The same standards for ethics, copyright, attributions and permissions as for the article apply. Supplements are not edited by Eurosurveillance and the journal is not responsible for the maintenance of any links or email addresses provided therein.

1. What Field Epidemiology Training Program (FETP) are you currently in?
   - ☐ The European Programme for Intervention Epidemiology Training (EPIET)
   - ☐ Canadian Field Epidemiology Program (CFEP)
   - ☐ US-CDC Epidemic Intelligence Service

2. What year of your Field Epidemiology Training Program (FETP) are you currently in?
   - ☐ First year
   - ☐ Second year

3. Are you using AI for your work as a FETP fellow?
   - ☐ Yes
     - If "Yes", then participants move to question 5
   - ☐ No
     - If "No", then participants move to question 4

4. Please describe why you do not currently use AI in your work.
   - Once answered, then participants move to question 14

5. How frequently do you currently use AI for your work?
   - ☐ Daily
   - ☐ Weekly
   - ☐ Monthly
   - ☐ On occasion

6. How comfortable are you using AI for your work?
   - ☐ Very comfortable
   - ☐ Comfortable
   - ☐ Somewhat comfortable
   - ☐ Uncomfortable
   - ☐ Very uncomfortable

7. As a FETP fellow, what are you using AI for? (check all that apply)
   - ☐ Write code
   - ☐ Troubleshoot coding errors
   - ☐ Draft text as a starting point for abstracts and other FETP written deliverables
   - ☐ Generate epidemiological study questions
   - ☐ Generate suggestions for analytic methods
   - ☐ Gather background information on a public health (or other) topic
   - ☐ Generate images for presentations

- ☐ Work efficiencies (e.g., drafting emails, note taking)
- ☐ Others, please specify: ___________________________

8. As a FETP fellow, what AI platforms are you using (check all that apply)?
   - ☐ ChatGPT (Open AI)
   - ☐ Claude (Anthropic)
   - ☐ Microsoft Bing Co-Pilot
   - ☐ Microsoft LM
   - ☐ LLaMA 3.1(Meta)
   - ☐ Mistral
   - ☐ Institution-specific AI platform (e.g., CDC Chatbot)
   - ☐ Gemini 1.5 (Google) / Perplexity
   - ☐ Others, please specify: _____________________

9. Briefly describe how AI has impacted your work.

10. Are there any barriers to using AI in your current role as a FETP fellow?
    - ☐ Yes
      - If "Yes", then participants move to question 11
    - ☐ No
      - If "No", then participants move to question 12

11. Please describe these barriers.

12. Have you encountered any ethical concerns, or data management and privacy issues while using AI in your work?
    - ☐ Yes
      - If "Yes", then participants move to question 13
    - ☐ No
      - If "No", then participants move to question 14

13. Please specify the ethical concerns, or data management and privacy issues you've encountered.

14. Does your institution have an AI policy or guideline?
    - ☐ Yes
      - If "Yes", then participants move to Question 15
    - ☐ No
      - If "No", then participants move to Question 17
    - ☐ I don't know if my institution has a policy for the use of AI
      - If "Don't know", then participants move to Question 17

15. Do you know where to find your institution's policy or guideline on the use of AI?.
    - ☐ Yes
      - If "Yes", the participants move to Question 16
    - ☐ No
      - If "No", then participants move to Question 17

16. Please provide the link to the policy or guideline (if public), or alternatively, if you could email a copy of the policy or guideline to [redacted]

17. Have you received formal or informal training in the use of AI (regardless of who provided the training)?
    - ☐ Yes
        - If "Yes", then participants move to Question 18
    - ☐ No
        - If "No", then participants move to Question 19

18. Please provide details of the training you received.

19. What type of AI training or resources would you like to receive?

20. Is there anything else you would like to share about your use of AI?
